# Green analytical chemistry and experimental design: a combined approach for the analysis of zonisamide

**DOI:** 10.1186/s13065-023-00942-1

**Published:** 2023-04-17

**Authors:** Soha G. Elsheikh, Abeer M. E. Hassan, Yasmin M. Fayez, Sally S. El-Mosallamy

**Affiliations:** 1 Analytical Chemistry Department, Faculty of Pharmacy, October 6 University, Giza, Egypt; 2 Analytical Chemistry Department, Faculty of Pharmacy, Cairo University, Kasr El Aini, Cairo, 11562 Egypt

**Keywords:** Experimental design, Green Analytical chemistry, Stability indicating method, Zonisamide

## Abstract

Green analytical chemistry principles, as well as experimental design, are a combined approach adopted to develop sensitive reproducible stability indicating HPLC method for Zonisamide (ZNS) determination. The optimal conditions for three chromatographic parameters were determined using a central composite design of the response surface. Kromasil C_18_ column (150 mm × 4.6 mm, 5 µm) was utilized with ethanol, H_2_O (30:70 v/v) as a mobile phase at a flow rate of 1 mL/min at 35 °C. Good reproducibility and high sensitivity were achieved along (0.5–10 µg/mL) concentration range. In contrast, the TLC-densitometric method was performed on aluminum plates precoated with silica gel 60F_254_ as a stationary phase and chloroform: methanol: acetic acid (8:1.5:0.5 by volume) as a developing system. Reproducible results were obtained in the range of (2–10 μg/band). The chromatograms of HPLC and TLC were scanned at 280 nm and 240 nm, respectively. The suggested methods have been validated following ICH guidelines, and no statistically significant differences were detected between the results of the current study and the official USP method. It was also found that using experimental design implements the green concept by reducing the environmental impact. Finally, Eco-Scale, GAPI and AGREE were used to assess the environmental impacts of the suggested methods.

## Introduction

Green chemistry was implemented by Paul Anastas, which was designated to substitute hazardous solvents with less or non-toxic ones [1]. Substituting methanol or acetonitrile with ethanol or water as green solvents were adopted in pharmaceutical analysis, particularly in chromatographic techniques [2]. Furthermore, eco-friendly mobile phases meet the requirements of the US Environmental Protection Agency for reducing the hazardous environmental effects of analysis wastes [3].

In compliance with green analytical chemistry principles recently, application of experimental design in developing and optimizing chromatographic methods has taken a significant concern. The ability of experimental design to simultaneously analyze multiple variables and their effects qualifies it as economical and environmentally friendly approach, as it reduces the experiments and hence consumption of solvents in accordance with achieving separation of drugs in combination or the presence of its degradation products as well as impurities [4–9].

**Fig. 1:**
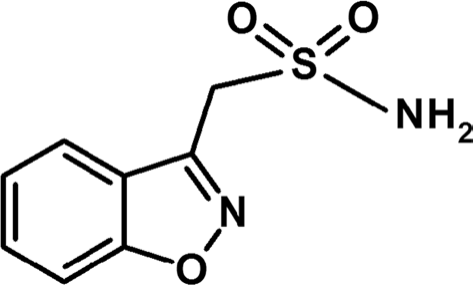
Chemical structure of Zonisamide

Zonisamide is a USP-approved medication with IUPAC name 1, 2-benzoxazol-3-yl methanesulfonamide (Fig. 1) [10], which is a member of the methanesulfonamide family with anticonvulsant properties. ZNS suppresses tonic–clonic as well as partial seizures in humans, in addition to preserving neurons from the damage induced by free radicals [11, 12].

By reviewing the literature, different methods were reported for determining ZNS either in pharmaceutical dosage form or biological fluids utilizing HPLC [13–18], capillary electrophoresis [19, 20], HPTLC [21, 22], and electroreduction at hanging mercury drop electrode [23]. To date, only a few stability-indicating methods were published for ZNS determination in the presence of its degradation products using HPLC [24–28] and spectrophotometric techniques [26, 29].

In this manuscript, the ZNS degradation pathway was demonstrated, and the degradation product was isolated, besides elucidating its structure utilizing IR and mass spectroscopy.

This study aims to develop and optimize simple, precise, and robust stability-indicating chromatographic methods that can be easily applied in the routine quantitative ZNS analysis. In addition to, HPLC method assisted with the Central Composite Design of the response surfaces to optimize the method so the number of experiments is minimized to achieving ecofriendly, time and cost-saving method.

TLC densitometry excels other methods as it enables rapid analysis as various samples can be tracked all together in the same time. Uses simple procedure and it is cost effective where small amount of solvent are consumed. The mentioned characteristics of both suggested methods are in line with the global trend toward application of ecofriendly techniques.

## Experimental

### Apparatus and software

The thin-layer chromatography (TLC) system consists of 100 μL Camag micro syringe (Muttenz, Switzerland) as well as Camag Linomat autosampler (Camag, Switzerland). TLC was performed utilizing 0.25 mm thickness TLC plates pre-coated with silica gel G.F254 20X20 cm (Merck, Darmstadt, Germany), winCATS software, and a short wavelength (254 nm) ultraviolet (UV) lamp (USA).

The HPLC system consists of Waters Alliance 2695 Separations Module using inline vacuum degassing with different flow rates, an autosampler, and programmable temperature control. A heated column compartment provides temperatures from 5 degrees above ambient to 65 °C. The wavelength range of model 2996 photodiode array detector is 190–800 nm. Stationary phase was Kromasil^®^ MS C_18_ column (150 mm × 4.6 mm, 5 µm) (Nouryon, Bohus, Sweden).Whatman syringe filter (Fisher, Nederland) was utilized. For IR and mass spectrometric analysis, an infrared (IR) spectrometer (Shimadzu, Japan) and an Ultra-performance liquid chromatography-mass spectrometer (LC/MS/MS) (Waters, Milford, USA) were used. Design-Expert software was used for data analysis (version 12) (Godward St NE, Minneapolis). Hot plate and pH meter were used (Jenway, Stone, UK).

### Chemicals and reagents

- Methanol and ethanol HPLC grade (Sigma Aldrich, MO, USA).

All other chemicals utilized in this work were of analytical grade.

- Analytical grade Chloroform (Merck, Germany).
- Acetic acid and Hydrogen peroxide (Al—Gomhoria Company, AZ Zaytoun Al Qebleyah, Cairo, Egypt).
- In-house preparation of double-distilled water was performed utilizing the AQUATRON water still A8000 system (Stone, Staffordshire, UK).
- Zonisamide was obtained from Mash premiere pharmaceuticals (Cairo, Badr city, Egypt), its purity was 99.87 ± 0.81 as per the USP official method [10].
- Convagran^®^ Hard gelatin Capsules, Batch No. M2012417 labeled to contain 25 mg/Capsule, Batch No. M2013617 labeled to contain 50 mg/Capsule, and Batch No. M2000918, labeled to contain 100 mg/Capsule, manufactured by Mash premiere Pharmaceutical (Badr City, Egypt), were bought from a local market.
- The degradation product was performed as detailed in the procedures.

### Standard solutions

- Zonisamide stock standard solution (1 mg/mL) prepared in methanol.
- The degradation product's stock standard solution (Equivalent to 1 mg/mL ZNS)*,* as illustrated in the procedures section, was prepared and quantitatively transferred into a volumetric flask of (25 mL), and then the volume was reached to the mark with methanol.
- *Working solutions for the HPLC method*

In order to obtain 100 µg/mL of working standard solutions in methanol, 10 mL of standard ZNS stock solutions, as well as the degradation product, were separately transferred into two volumetric flasks (100 mL/each).

### Procedures

#### Zonisamide degradation product preparation

From ZNS pure powder, 25 mg were weighed and transferred into 25 mL volumetric flask. The minimum amount of methanol was then added for dissolving ZNS. The volume was reached the mark with 10% H_2_O_2_ and the solution was refluxed on a hot plate at 80 °C for 16 h. TLC aluminum sheets were utilized to assure complete sample degradation every two hours, using chloroform: methanol: acetic acid (8:1.5:0.5 by volume) as a developing system. The fully degraded solution was subsequently evaporated to dryness, cleansed by dissolving in methanol and re-evaporated multiple times. Finally, IR and mass spectroscopy were utilized to elucidate degradation product structure.

#### Calibration curve of TLC-Densitometry method

Application of various aliquots from the ZNS stock solution was performed on the TLC plates and developed up to 8.5 cm using developing system [chloroform: methanol: acetic acid (8:1.5:0.5 by volume)]. The plates were air-dried, and the spots were visualized at 254 nm under a UV lamp. Chromatograms were scanned at 240 nm. The polynomial equation was derived from a calibration curve depicting the correlation between integrated peak area and the corresponding ZNS concentrations in range of (2-10 µg/band).

#### Calibration curve of HPLC method

Five to one hundred micrograms of the working ZNS standard solution (100 µg/mL) were transferred separately into a set of volumetric flasks (10 mL/each); the volume was completed until reaching the mark with the mobile phase, and each concentration was repeated in triplicate. Samples were filtered utilizing syringe filters, and the separation was performed on Kromasil^®^ C_18_ column (150 mm, 4.6 mm, i.d.5 μm) using ethanol: H_2_O (30:70 v/v) as mobile phase with 10 µL injection volume, at flow rate of (1 mL/min) and temperature was adjusted to be 35 °C. At 280 nm, chromatograms were observed. After constructing the calibration curve via plotting the peak area against the corresponding concentrations of ZNS, the linear regression equation was determined.

#### Application to pharmaceutical formulations

Ten hard gelatin capsules of Convagran^®^ from each of the three dosage forms were emptied, and each powder was thoroughly mixed. A stock solution for each dosage form claimed to contain (1 mg/mL) ZNS was prepared by weighing the accurate amount from each powder into three suitable separate volumetric flasks. The powder was dissolved with methanol, sonicated for 20 min, and then filtered.

For the TLC method: Different aliquots from each dosage form stock capsule solutions were spotted on the TLC plates.

For HPLC method: The working solution for each dosage form was prepared by diluting 10 mL of stock solutions into three separate volumetric flasks (100 mL/each). Aliquots were transferred from working solutions into three separate series of volumetric flasks (10 mL/each), and the volume was brought up to the mark with the mobile phase.

## Results and discussion

Solvents in the pharmaceutical field are more than half of the material used in the manufacturing process or the analysis of their finished products. Several environmental organizations and agencies worldwide strive to conserve the environment against human misuses, such as Environmental Protection Agency and Global Environment Facility. One of their solutions to attain their goals of reducing economic and environmental losses; is to use green solvents. Therefore, the present study aimed to use green solvents while determining zonisamide in the presence of its degradation product.

### Zonisamide degradation

Submission of the accelerated stability studies [6 months] and late stability studies [3 years] for Governmental agencies regulating medication are mandatory in the registration of a new drug for manufacture. In the pharmaceutical industry, stability studies are considered in order to ensure that no changes will occur in the concentration of the active ingredients and that no toxic degradation products will be formed, as well as to determine the optimal storage conditions [30, 31]. ZNS breakdown when refluxed with 10% H_2_O_2_ for 16 h at 80 °C of continuous heating. Spot of different R_*f*_ values was observed for the oxidative degradation product and confirmed by examining the solution every two hours on TLC plates using chloroform: methanol: acetic acid (8:1.5:0.5 by volume) as a developing system which proved complete degradation. It is worth mentioning that ZNS showed stability towards different concentrations of HCl and NaOH (0.1N, 1N, 2N, and 5N) at varying temperatures.

**Fig. 2:**
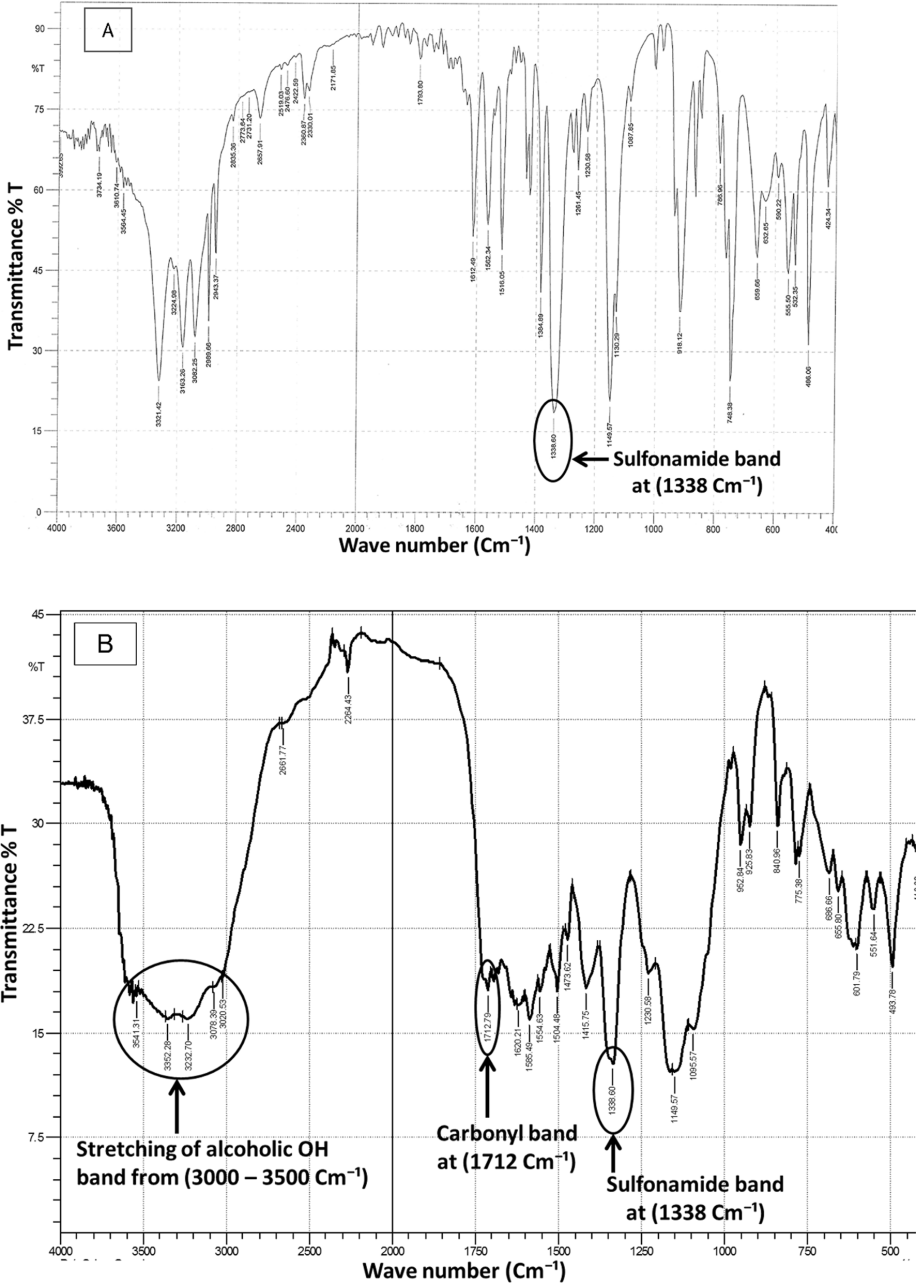
**A** IR spectra of intact ZNS and **B** IR spectra of degradation product

Infrared (IR) and mass spectroscopy, were utilized for elucidating the degradation product structure. IR spectrum of pure ZNS (Fig. 2A) indicates the existence of a characteristic band at 1338 cm^−1^ corresponding to the sulfonamide group, which is still apparent in the degradation product IR spectrum. Furthermore, the existence of a band at 1712 cm^−1^ indicates the presence C = O and a broad band at 3000 to 3500 cm^−1^ indicates the existence of alcoholic OH group in the degradation product's IR spectrum (Fig. 2B).

**Fig. 3:**
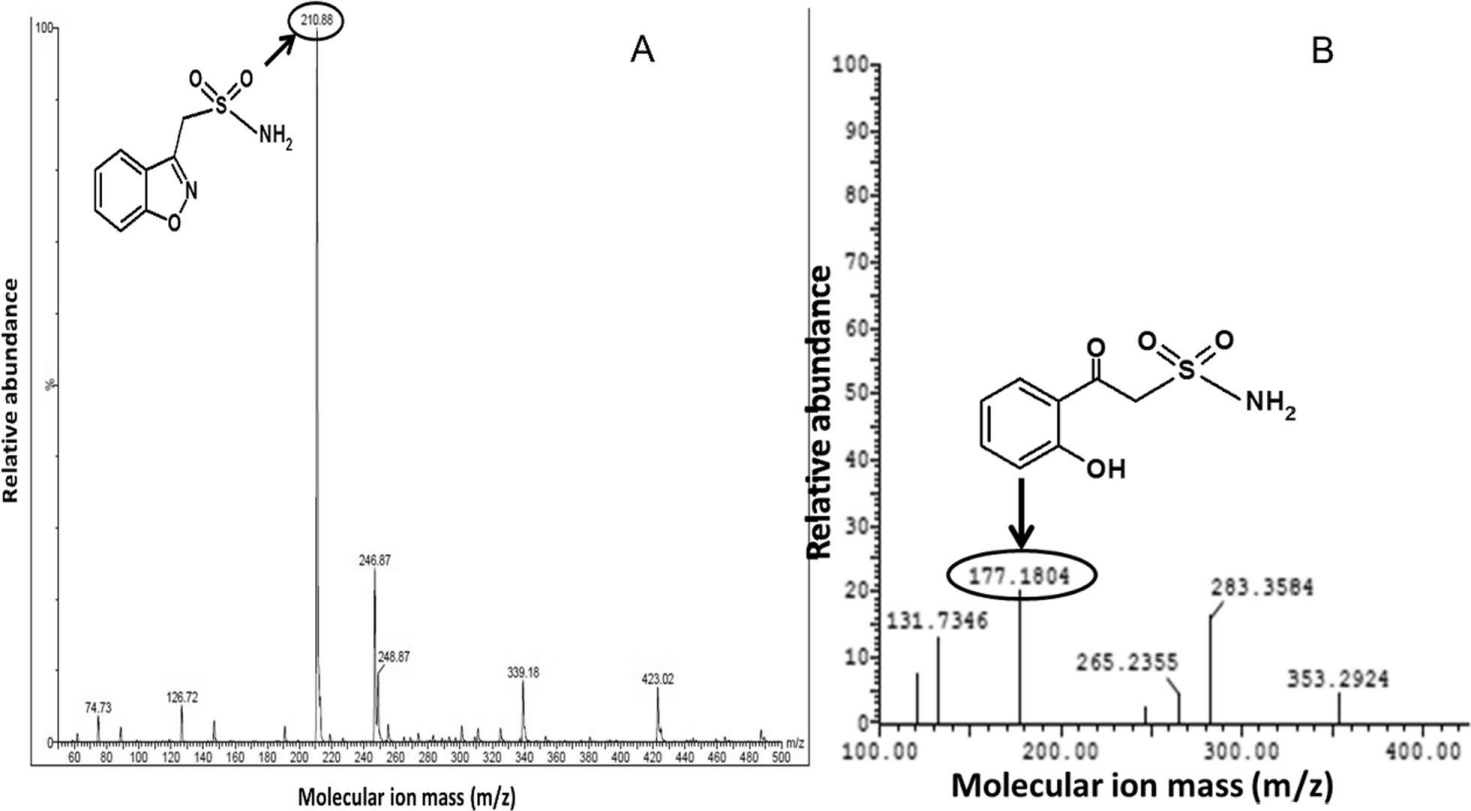
**A** Negative ESI–MS of zonisamide, **B** Positive ESI–MS of zonisamide oxidative degradation product

**Fig. 4:**
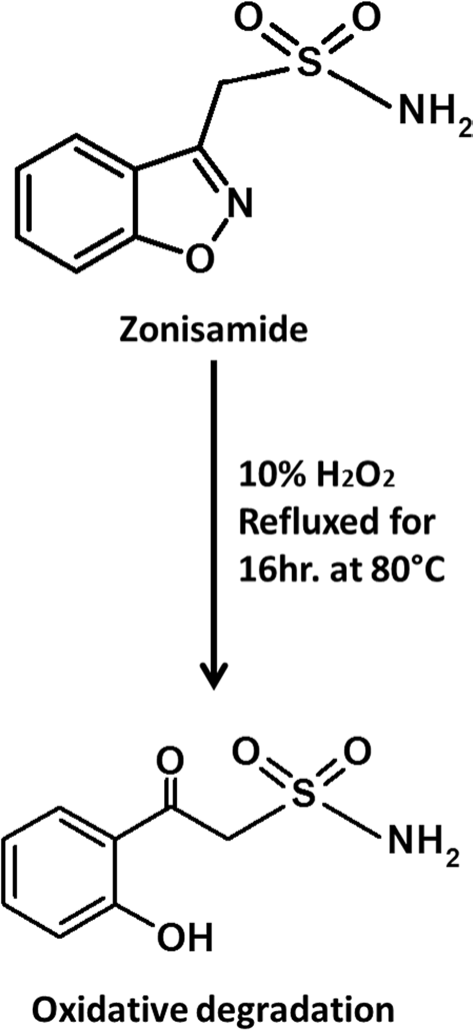
Suggested pathway for oxidative degradation of zonisamide

The negative ESI–MS scan demonstrated a ZNS molecular ion peak at (m/z = 210.88) (Fig. 3A) while (m/z = 177) for the ZNS degradation product of the positive ESI–MS scan (Fig. 3B). Accordingly, the oxidative degradation pathway ZNS was expected, as shown in (Fig. 4).

### TLC Method

**Fig. 5:**
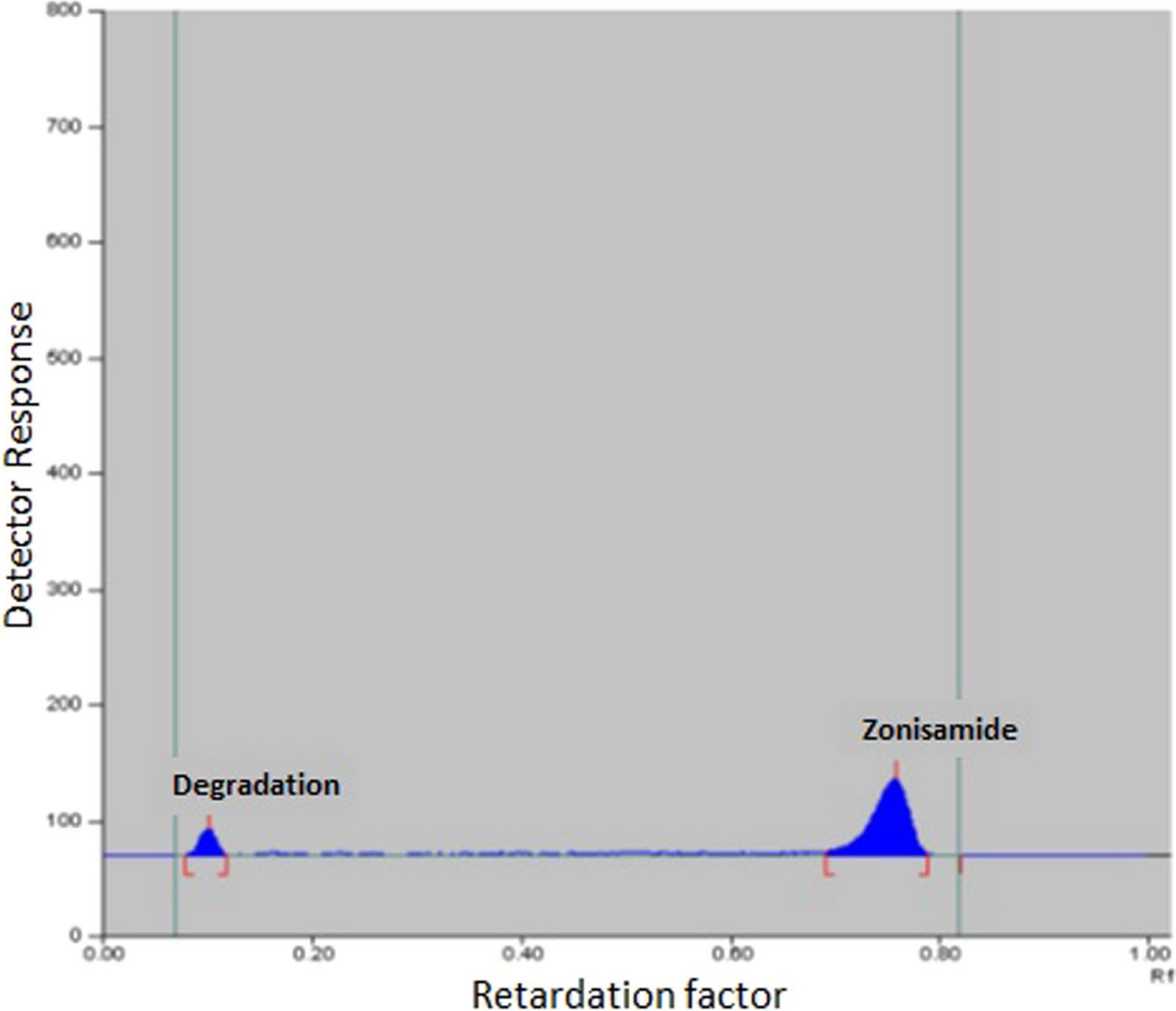
TLC densitogram of Zonisamide (R_*f*_ = 0.76) and its degradation product (R_*f*_ = 0.11) at 240 nm

Zonisamide was estimated in the presence of its degradation product using the densitometric method after a number of preliminary trials. These trials were performed utilizing various developing systems in varying ratios until determining the optimal conditions with promising results. A tailed degradation product's peak was observed using the following developing systems [ethyl acetate: acetic acid] and [butanol: propanol]. A poor resolution was obtained upon using [methanol: butanol: chloroform] and [propanol: butanol: chloroform]. Different ratios of [chloroform: methanol: acetic acid] have been experimented as developing system. Multiple attempts to reduce chloroform and methanol volume were investigated in order to minimize their harmful impact. No separation was observed when the volume of chloroform is lower than 8 mL. The results were satisfactory upon using ratio of (8:1.5:0.5 by volume) where the degradation product spot is no longer retained at the baseline. At 240 nm, the densitogram was scanned, and R_*f*_ values were 0.76 for ZNS and 0.11 for its degradation product (Fig. 5). There is a polynomial correlation between the integrated peak area and the corresponding ZNS concentration ranging between (2 to 10 µg/band) at 240 nm, which was the wavelength of choice where both ZNS and its degradation demonstrate good sensitivity.

### HPLC Method

In our work, preliminary trials were conducted to determine the optimal column type, length, and temperature, as well as the optimal ratio of the green mobile phase. Cyano and C_18_ columns were initially evaluated in order to select the one that progressively improve the system suitability parameters. Cyano column showed poor resolution with tailing peak for both analytes; in contrast to C_18_. Different temperatures) 25, 30, and 35 °C( were tried and no significant effect was observed on retention time, but the bifid peak shape was overcome at temperature ˃ (25 °C). Different ratios of [ethanol: H_2_O] were tried and it has been noticed that upon increasing water percentage, the retention time increased.

Regular practice during developing HPLC methods usually relies on studying each variable and its effect separately, which is time and cost-consuming [32, 33]. This paper refers to the application of a central composite of surface response design of expert that has the advantages of studying various variables simultaneously, in addition to optimization of HPLC conditions. Accordingly, the time and cost consumption will be decreased [34–39]*.*

**Fig. 6:**
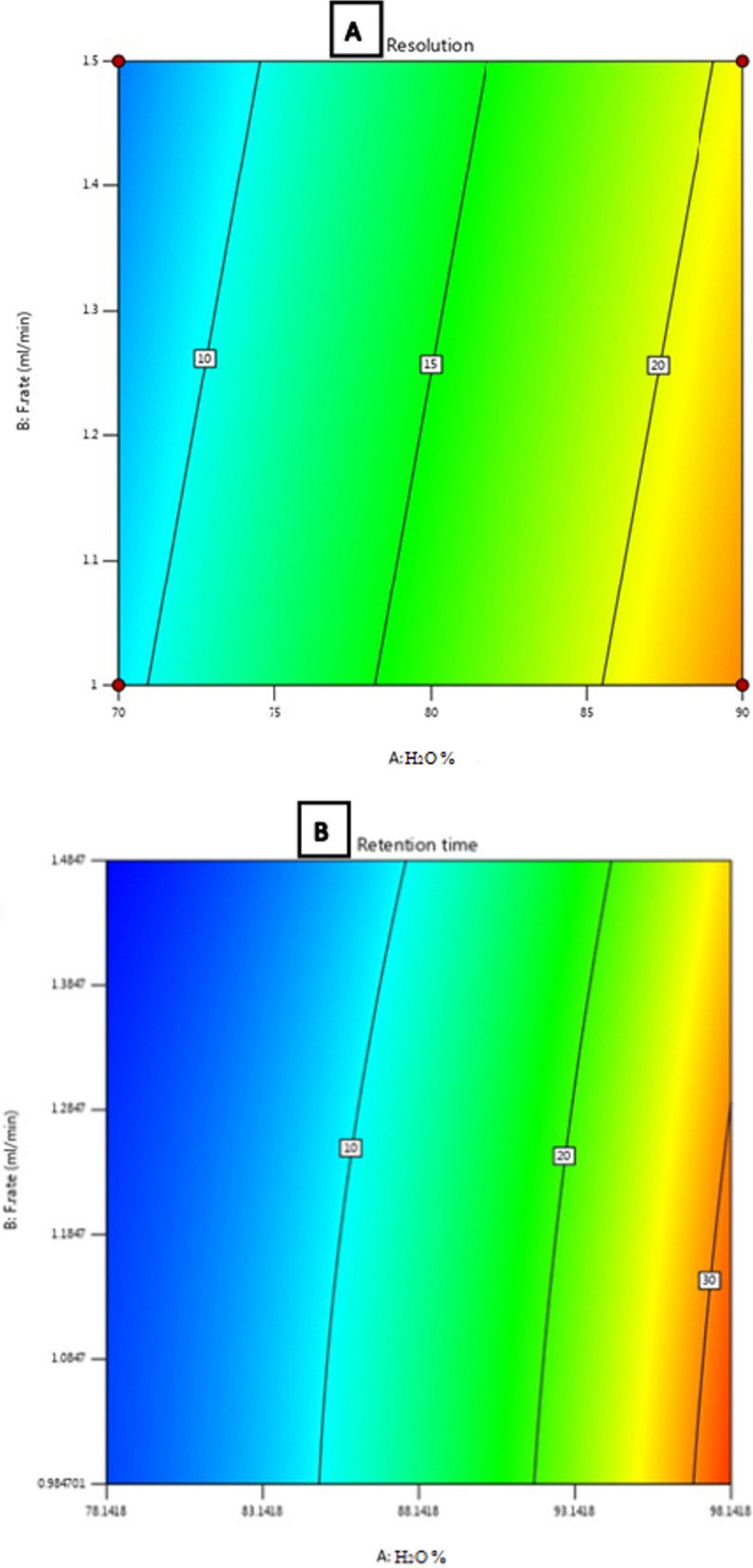
Contour surface diagrams showing the effects of water % and flow rate on **A** Resolution (Rs) and **B** Retention time (Rt)

**Fig. 7:**
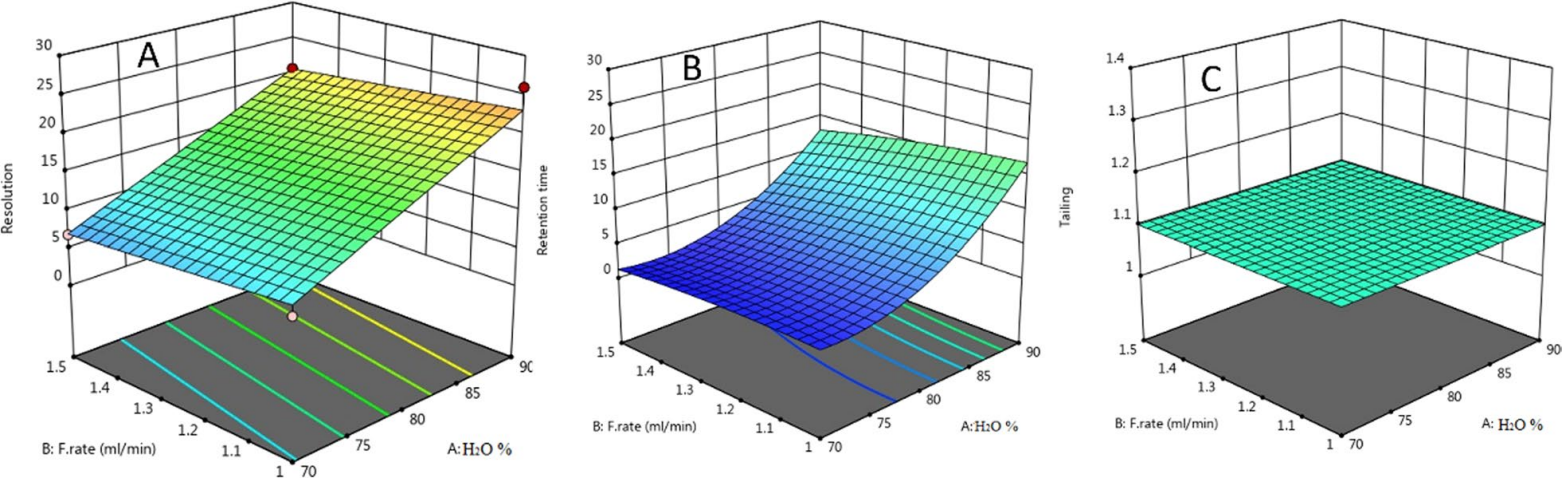
The 3D surface response plot of desirability for optimization of factors **A** Resolution (Rs), **B** Retention time (Rt) and **C** Tailing (T)

This work applied experimental design to investigate three factors and analyze their effect simultaneously. Mobile phase ratio, flow rate, and temperature were three factors significantly affect the HPLC response. The experimental design is based on two factorial levels coded with [−1] for each factor's low level and [+ 1] for the high level. According to preliminary studies results, the ratio of the mobile phase in the low level was 70% and in the high level was 90%; however, the flow rate was [1 mL/min] for low and [1.5 mL/min] for high. Finally, the temperature factor is 25 °C for low level and 35 °C for high level. The design of expert software suggested a total of twenty runs, including six center point runs, to examine the effect of each factor on resolution, retention time, and tailing factor. The objective of the statistical analysis using Derringer's desirability function was to achieve maximum resolution with the shortest retention time. Maximum resolution value was attained at a higher percentage of water (90%) and 1 mL/min flow rate (Fig. 6A). However, the lowest retention time was achieved at the percentage of water in mobile phase (~ 60%) and 1.25 mL/min flow rate (Fig. 6B). There was no significant effect observed on tailing value, as demonstrated in (Fig. 7).

**Fig. 8:**
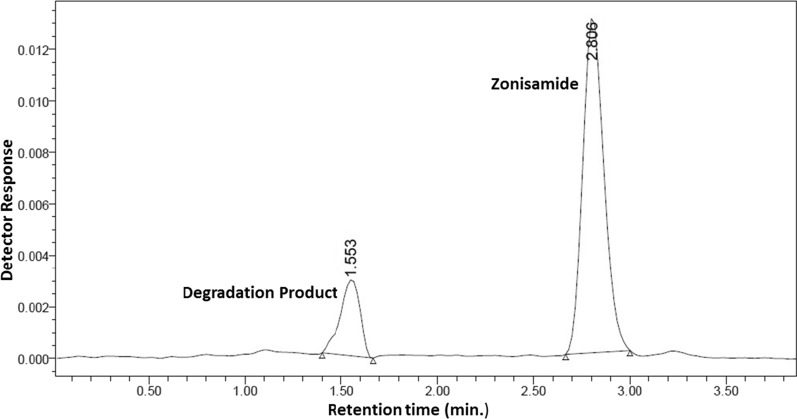
HPLC chromatogram of ZNS of 50 µg/ml (tR = 2.806) and its degradate of 10 µg/ml (tR = 1.553) at 280 nm

As suggested by the experimental design, the optimal ZNS chromatographic separation from its degradation product was achieved utilizing Kromasil^®^ MS C_18_ (150 mm, 4.6 mm, i.d.5 μm) column at 35 °C with green as well as eco-friendly mobile phase containing [ethanol: H_2_O (30:70 v/v)], at (1 mL/min). Linear correlation between the peak area the corresponding ZNS concentration ranging between 2 to 10 µg/mL was scanned at 280 nm (Fig. 8), and the regression equation was determined as A = 14327C − 300.15 r = 1, where A represents the peak area, r is the correlation coefficient, and C is the concentration in μg/mL.

The results of system suitability parameters revealed that the proposed system is adequate for analyzing ZNS and the parameters calculated per the USP guidelines (Table 1) [10]. In addition, satisfactory results for validation parameters were presented for both suggested methods in (Table 2) according to ICH guidelines (Q2) R1 recommendations [40]. The proposed methods were effectively guaranteed for the analysis of ZNS in Convagran^®^ capsules, and the methods was further validated via the application of the standard addition technique (Table 3). Additionally, a statistical comparison of the results obtained from the pharmacopeia HPLC method [10] and the proposed methods showed no significant differences regarding precision, and accuracy (Table 4).

**Table 1:** Parameters required for system suitability tests of TLC–densitometric and HPLC methods

Method	TLC		HPLC		Reference values [12]
Parameter	Zonisamide	Degradation product	Zonisamide	Degradation product	
t_R_ (min)	–	–	2.806	1.553	–
R_f_	0.76	0.11	–	–	–
Resolution (R_s_)	14.38		6		R_s_ > 2
Tailing factor (T)	1	0.9	1	0.83	≈1
Capacity factor (K)	0.31	9	4.61	2.11	0–10
Selectivity factor (α)	7.6		2.18		α > 1
Column efficiency (N)	–	–	3831.26	866.41	Increase with efficiency of the separation
Height equivalent to theoretical plates(HETP) (mm)	–	–	0.03	0.12	The smaller the value the higher the column efficiency

**Table 2:** Assay and validation parameters for the determination of ZNS by the proposed methods

Parameter	TLC	HPLC
Range	2–10 µg/band	0.5-10 µg/mL
Slope^a^	− 83.851^*^ 2094.5^**^	14,327
Intercept	5987.4	− 300.15
Correlation coefficient (r)	0.9997	1
Accuracy	99.97 ± 1.22	100.15 ± 0.52
Repeatability ^b^	1.04	0.77
Intermediate precision ^c^	0.94	0.88
Robustness ^d^	1.28	1.21
LOD	0.63 µg/band	0.07 µg/mL
LOQ	1.90 µg/band	0.21 µg/mL
SE of Intercept	46.00	251.73
SE of slope	15.21	41.55

^a^Slope * and ** are the coefficients of *X*^*2*^ and *X*, respectively. Following a polynomial regression *y* = *ax*^*2*^ + *bx* + *c* Where, *A* is the peak area, *x* is the concentration of ZNS (μg/band), *a* and *b* are coefficients * and **, respectively and c is the intercept

^b^RSD (%) of three concentrations of ZNS (3, 5 and 7 µg/band for TLC) and (4, 6 and 8 µg/mL for HPLC) analyzed intra-daily in triplicate

^c^RSD (%) of three concentrations of ZNS (3, 5 and 7 µg/band for TLC) and (4, 6 and 8 µg/mL for HPLC) analyzed inter-daily on three successive days

^d^RSD% of ZNS at three different ratio mobile phases of ethanol: H_2_O (30:70 ± 2 mL of H_2_O) for HPLC and chloroform: methanol: acetic acid (8: 1.5: 0.5) ± 0.5 mL of methanol) for TLC

**Table 3:** Determination of ZNS in its pharmaceutical form and application of standard addition technique results using the proposed methods

Convagran^®^ capsules 25 mg ZNS/capsule B.N. M2012417			TLC	HPLC
Found^*^% ± SD			99.84 ± 0.25	100.64 ± 0.84
Standard addition technique	Taken^*^	Added^*^	Recovery%	
	4	2	101.76	99.98
		4	100.88	98.93
		6	100.73	99.68
	Mean ± SD		101.12 ± 0.56	99.53 ± 0.54

Convagran^®^ capsules 50 mg ZNS/capsule B.N. M2013617			TLC	HPLC
Found^*^% ± SD			100.79 ± 0.88	99.51 ± 1.11
Standard addition technique	Taken^*^	Added^*^	Recovery%	
	4	2	100.24	99.67
		4	100.20	98.97
		6	100.94	100.40
	Mean ± SD		100.46 ± 0.42	99.68 ± 0.72

Convagran^®^ capsules 100 mg ZNS/capsule B.N. M2000918			TLC	HPLC
Found^*^% ± SD			100.02 ± 1.36	99.69 ± 0.68
Standard addition technique	Taken^*^	Added^*^	Recovery%	
	4	2	100.72	98.52
		4	100.76	100
		6	100.63	100.69
	Mean ± SD		100.71 ± 0.07	99.74 ± 1.11

^*^Taken concentration for TLC = (4 µg/band), while added concentration = (2, 4, 6 µg/band) and for Taken concentration HPLC = (4 µg/mL), while added concentration = (2, 4, 6 µg/mL)

**Table 4:** Statistical comparison between the results obtained by the proposed methods and the pharmacopeial method for the analysis of ZNS

	TLC	HPLC	Official method^*^
Mean	99.97	100.15	99.87
SD	1.22	0.52	0.81
Variance	1.50	0.27	0.65
N	5	6	7
Student’s t-test^**^	0.172 (2.23)	0.728 (2.20)	
F value^**^	2.31 (4.53)	2.41 (4.95)	

^*^Official HPLC method using C18 column, acetonitrile: methanol: 1.36 g/L phosphate buffer (1:1:8 by volume) as a mobile phase and UV detection at 240 nm

^**^Figures between parentheses represent the corresponding tabulated values of t and F at *p* = 0.05

To the best of our knowledge, the proposed TLC method is the first for determining ZNS along with its degradation product. The HPLC proposed method has an advantage over other published methods [41, 42], as it is the first methodology to determine ZNS in the presence of its degradation product using green analysis coupled with an Experimental Design, which in turn decreases the number of trials performed hence making it the most economical and the least time-consuming method. Accordingly, the retention time of ZNS in the present study (2.8 min.) is the shortest run time compared to that reported by previous similar studies.

High reproducibility and ecofriendly are advantages of the proposed HPLC method over the proposed TLC- densitometry method. However, Procedure simplicity and rapid analysis were possessed using the proposed TLC- densitometry by spotting numerous samples on one plate and developing all together at the same time.

In the current HPLC method, solvents with hazardous effects on the environment were replaced with ethanol and water to avoid safety concerns, and adverse health effects. Recently, the global trend is directed to replace harmful solvents with safer ones [43]. Registration, Evaluation, Authorization, and Restriction of Chemicals regulation and the European Chemicals Agency encourage using green solvents to minimize the negative ecological and economic outcomes of hazardous solvents and their toxic wastes [44]. Regarding the effect of the methods on the environment, Eco-Scale, GAPI and AGREE tools indicate that these two suggested methods are considered more ecofriendly than pharmacopeia HPLC method [10].

Firstly, the Eco-Scale tool evaluates the greenness of the analytical method, with a score of 100 corresponding to the ideal method. Greenness assessment of method achieved via penalty points as excellent green analysis determined with score > 75 while acceptable with score > 50 and inadequate analysis with score < 50 [45]. The calculated scores for the proposed methods, (84) and (91) for the TLC-densitometry and the HPLC methods, respectively (Table 5).

**Table 5:** Penalty points of greenness assessment of the chromatographic suggested methods by Eco-Scale

Reagents/Instruments	Proposed TLC method	Proposed HPLC method	^*^Official HPLC method [9]
Ethanol		4	
Methanol	6		6
Phosphoric acid			2
Chloroform	4		
Acetic acid	2		
acetonitrile			4
TLC	1		
HPLC		1	1
Occupational hazard	0	0	0
Waste	3	4	6
Total penalty points	Ʃ 16	Ʃ 9	Ʃ 19
Analytical Eco-Scale total score	84	91	81

^*^Official HPLC method using C18 column, acetonitrile: methanol: 1.36 g/L phosphate buffer (1:1:8 by volume) as a mobile phase and UV detection at 240 nm [9]

Secondly, low hazardous impact of the developed methods via GAPI has been demonstrated. The results are illustrated by color scale pictogram consisting of five pentagrams which include; sample preparation, reagent and solvent, as well as instrumentation. Green color refers to much safer impact to the nature, yellow means problematic impact while the red color refers a hazardous impact to the nature and preferred to be avoided. Pictogram of the proposed HPLC method resulted in 9 green colors, 2 red and 4 yellow while the proposed TLC pictogram resulted in 10 green colors, 3 red and 2 yellow, which representing greener more than the official one as shown in (Table 6).

**Table 6:** Greenness assessment of the chromatographic proposed methods by GAPI and AGREE

Method	GAPI	AGREE
Official method [9]		
Proposed HPLC		
Proposed TLC -densitometry		

Finally, the greenness assessment of the proposed chromatographic methods was conducted using AGREE tool. Twelve principles have been applied using greenness calculator software that achieves a clock like graph. AGREE pictogram demonstrating a score in the middle with color grading from intense green to intense red that illustrated the eco-friendly impact. AGREE pictograms of the developed HPLC and TLC-densitometry methods show a score of (0.75) and (0.76) with faint green colors, respectively. The results of the comparison between the obtained method and the official USP method [10] shown in (Table 6) prove that the suggested methods were more ecofriendly. Despite the greenness scores of ZNS official method and their proximity to the suggested ones; it was observed that the official method remains to have a negative impact consistent with Environment, Health and Safety [EHS] [46], as pharmacopeia HPLC method use acetonitrile in its mobile phase. In addition, using various buffers consumes time and chemicals during preparation steps. According to the list ranked by the American Chemical Society Green Chemistry Institute^®^, the mobile phase of the proposed HPLC method constitute two of the top three green solvents [43, 47].

## Conclusion

Zonisamide was only sensitive to oxidation degradation. To date, the degradation pathway and structure of the degradation product haven’t been demonstrated by IR or mass spectrometry. The suggested chromatographic methods for the routine analysis of ZNS have been demonstrated to be valid and efficient. The greenness assessment of the suggested methods revealed their superiority over the official one. By studying and optimizing the impact of three distinct variables simultaneously, independently, and in combination, the experimental design achieved a crucial objective in optimizing the simple, rapid, and robust HPLC method.

## Data Availability

Datasets generated and/or analyzed during the current study are available from the corresponding author on reasonable request.
